# Supplementary material for: A genome-wide CRISPR screen identifies CALCOCO2 as a regulator of beta cell function influencing type 2 diabetes risk
Source: Nat Genet. 2022 Dec 21;55(1):54–65. doi: 10.1038/s41588-022-01261-2 (PMC9839450; doi:10.1038/s41588-022-01261-2)
Supplement: Supplementary file 1 — Supplementary Tables 2 and 3, Methods and Discussion. [file 41588_2022_1261_MOESM1_ESM.pdf]

nature genetics

Article https://doi.org/10.1038/s41588-022-01261-2

A genome-wide CRISPR screen identifies *CALCOCO2* as a regulator of beta cell function influencing type 2 diabetes risk

In the format provided by the authors and unedited

Supplementary Information

A genome-wide CRISPR screen identifies *CALCOCO2* as a regulator of beta cell function influencing type 2 diabetes risk

Antje K Rottner[1], Yingying Ye[2*], Elena Navarro-Guerrero[3*], Varsha Rajesh[2], Alina Pollner[2], Romina J Bevacqua[4,5], Jing Yang[2], Aliya F Spigelman[6], Roberta Baronio[3], Austin Bautista[6], Soren K Thomsen[1], James Lyon[6], Sameena Nawaz[1], Nancy Smith[6], Agata Wesolowska-Andersen[8], Jocelyn E Manning Fox[6], Han Sun[2], Seung K Kim[4,5], Daniel Ebner[2], Patrick E MacDonald[6], Anna L Gloyn[1,2,5,7,8#]

1. Oxford Centre for Diabetes, Endocrinology and Metabolism, Radcliffe Department of Medicine, University of Oxford, Oxford, OX3 7LE, UK.
2. Department of Pediatrics, Division of Endocrinology, Stanford School of Medicine, Stanford University, Stanford, CA, USA.
3. Target Discovery Institute, Nuffield Department of Medicine, University of Oxford, Oxford OX3 7FZ, UK.
4. Department of Developmental Biology, Stanford University School of Medicine, Stanford, CA, USA.
5. Stanford Diabetes Research Centre, Stanford School of Medicine, Stanford University, Stanford, CA, USA
6. Department of Pharmacology and Alberta Diabetes Institute, University of Alberta, Edmonton, Alberta, Canada
7. Wellcome Centre for Human Genetics, Nuffield Department of Medicine, University of Oxford, Oxford, OX3 7BN, UK.
8. Oxford NIHR Biomedical Research Centre, Oxford University Hospitals Trust, Oxford, OX3 7LE, UK.

*These authors contributed equally. *#Correspondence:* Anna L. Gloyn, Division of Endocrinology, Department of Pediatrics, Stanford School of Medicine, Stanford University, Stanford, CA, USA. agloyn@stanford.edu

Supplementary Tables

Supplementary Table S2 – Prioritized causal genes for T2D based on integration with T2D effector gene predictions.

Genome-wide CRISPR screen	eQTL for T2D (Viñuela et al. 2020)
AGPAT2	*ADCY5*
ARAP1	*AP3S2*
CALCOCO2	*CAMK1D*
DCUN1D4	*STARD10*
FADS1	*CEP68*
IGF2	*DGKB*
INS	*GPSM1*
IRS2	*HMG20A*
MCCC1	*PLEKHA1*
MED23	*ITGB6*
NKX2-2	*RNF6*
NUDT3	*TCF7L2*
PELO	*UBE2E2*
PPP1R15B	*NKX6-3*
RBMS1	*GRB14*
SHQ1	*HAUS6*
SIN3A	*IGF2BP2*
SLC2A2	*KLHL42*
TBC1D4	*ABCB9*
ZNF101	*SCD5*
	SLC12A8
	SLC7A7
	PDE8B

Common genes from effector transcript predictions and the genome-wide CRISPR screen (left) or from a recent human islet eQTL studies for T2D (right)[19].

Supplementary Table S3 – Human Tissue Donor Details.

Tissue source	*Sample Identifier*	*Sex*	*Age (yrs)*	*BMI (Kg/m^2)*
IIDP	SAMN18021384	Male	56	24.2
IIDP	SAMN18479112	Male	44	24.4
ADI	R398	Female	51	25.4
NDRI	OD30535	Female	31	48.2

Tissue samples for Immunostaining and functional shRNA experiments. IIDP, Integrated Islet Distribution Program (https://iidp.coh.org). ADI, Alberta Diabetes Institute (http://www.bcell.org/adi-isletcore.html). NDRI, National Disease Research Interchange.

Supplementary Methods

Cell culture

EndoC-βH1 were cultured in DMEM containing 5.5 mM glucose (Gibco), 2% bovine serum albumin (BSA), 2 mM glutamine, 10 mM nicotinamide, 100 international units (U)/ml penicillin, 100 µg/ml streptomycin (P/S), 50 µM β-2-mercaptoethanol, 5.5 µg/ml transferrin and 6.6 ng/ml sodium selenite in culture vessels coated with 2 µg/ml Fibronectin and 1% extracellular matrix (ECM) (all Sigma-Aldrich). The cells were passaged once a week and seeded at 48 000 cells/cm^2.

Cloning of individual sgRNAs

plentiCRISPRv2 was a gift from Feng Zhang (Addgene plasmid # 52961) and sgRNA sequences are listed in Supplementary Table 4. BsmBI compatible tails, 5'CACCGX3' and 5'AAACYC3', with X and Y being complementary sequences to the sgRNA, were added to each of the sgRNA oligonucleotides. Digestion of plentiCRISPRv2 vector was performed with FastDigest BsmBI (Fermentas) for 30 min at 37°C followed by gel-purification using a 0.8% agarose gel. sgRNA oligos were annealed (1 µl of each 100 µM stock) and phosphorylated using T4 PNK (NEB) by incubating them for 30 min at 37°C, 5 min at 95°C followed by cooling them down to room temperature by shutting off the heating block. Ligation of 20 ng of BsmBI digested plentiCRISPRv2 and 2 µl of 1:100 diluted annealed sgRNA oligonucleotides was performed using Quick Ligase (NEB) for 1 h at RT. Finally, 5 µl of the ligation reaction were transformed into Stbl3 competent cells and successful cloning was verified using Sanger sequencing.

Pooled sgRNA library amplification

To maintain library representation, four electroporation reactions were set up using 2 µl of 50 ng/µl TKOv3 library to 25 µl of Endura Competent Cells (Lucigen) each. The electroporation was performed following manufacturer's instructions followed by addition of 975 µl recovery medium (Lucigen). An additional 1 ml of recovery medium was added, cells were placed in a shaking incubator at 250 rpm for 1 h at 37°C and subsequently pooled. Full library representation was assessed based on a titer to estimate the transformation efficiency in parallel to the actual library

amplification. The titer was performed by adding 10 µl of cells to 990 µl of recovery medium, mixing and plating 20 µl onto pre-warmed 10 cm LB agar plates containing 100µg/ml carbenicillin (all Sigma-Aldrich) which is equivalent to a 40 000-fold dilution of the full transformation reaction. The library amplification was performed with 400 µl of the recovered cells by spreading them on one pre-warmed 15 cm LB and carbenicillin agar plate. All plates were incubated for 14-16 h at 30°C. The transformation efficiency was calculated by counting the number of colonies on the dilution plate and multiplying them by 40 000 to get the total number of colonies. Library amplification was continued if a coverage of 200 colonies per sgRNA was achieved which is equivalent to 1.4 x 107 colonies in the titer. The amplified library containing colonies were harvested by adding 7 ml of LB media containing 100µg/ml carbenicillin to each plate, scraping with a cell spreader followed by transfer and pooling in a centrifugation bottle. The transformed cells were centrifuged for 10 min at 5000 rpm and the wet pellets weights was measured to estimate the amount of plasmid purification columns. The library plasmids were extracted using the plasmid mega kit (Qiagen). sgRNA representation was confirmed by sequencing on a NextSeq500 (Illumina) using 75 base pair (bp) single end reads achieving a total mapped read depth of 22.7 million reads which corresponds to 319 mapped reads per sgRNA. 99.61% of all sgRNAs were detected with a low number of over- or underrepresented sgRNAs, indicating even and good library representation.

Lentiviral production and transduction

HEK293T cells were co-transfected with lentiviral packaging vectors in P/S free media (pMD2.G (6.85 µg) (Addgene #12259), psPAX2 (10.3 µg) (Addgene #12260), plentiCRISPRv2 with cloned sgRNA or library (12.85 µg), 2 ml of JetPrime buffer and 60 µl of JetPrime transfection reagent (Polyplus transfection) per 80% confluent T175 flask. The transfection mix was incubated for 15 min at RT and added to the media which was replaced after 16 h. 48 h after transfection, the supernatant containing virus was collected, centrifuged for 5 min at 2000 rpm and filtered through a 0.45 µm filter to remove cells and debris. Virus was collected through ultracentrifugation for 2 h at 4°C in a swinging-bucket rotor at 29000 rpm and resuspended in 1.5 % BSA in PBS.

Functional lentiviral titer

EndoC-βH1 were plated at 20,000 cells per well in a 96-well plate and virus was diluted from 1:50 to 1:6400 in 100µl P/S free media. Transduction was performed 48 h after plating and the cells were incubated with diluted virus for 6 h. After 48 h, media was changed on half of the wells into 4 µg/µl puromycin containing complete media. Cell viability was measured after 7 more days using the CyQUANT Direct Cell Proliferation assay (Invitrogen). Cell counts from puromycin selected wells were normalised to their respective non-selected controls to calculate the percentage of survival which is an estimate of successfully transduced cells. The functional titer in transducing units (TU)/µl can then be calculated using:

$$(1)\ TU/\mu l = \frac{\#\ \text{Cells} \times \text{m}}{\text{Virus (µl) used in transduction}}$$

The probability that a cell is infected by a certain number of viral particles at a given multiplicity of infection (MOI) (m) can be modelled using the Poisson distribution (PD). The original PD equation can be reduced to:

$$(2)\ P(n > 0) = 1 - e^{-m}$$

where P(n>0) is the probability that a cell gets infected by at least one viral particle. The MOI with a majority of cells only being infected by a single viral particle is an MOI of 0.3 which infects approximately 26 % of cells. The MOI relative to the virus (µl) used in transduction in Equation 1 was calculated by using a linear regression for the percentage of alive cells against the amount of infected virus in the linear, unsaturated range of the puromycin selection curve. Based on the known constants for an MOI of 0.3, the amount of virus needed was calculated by inserting 26 % as the percentage of surviving cells and solving the linear equation. Along with the number of plated cells, the TU/µl was then determined.

Supplementary Discussion

Our CRISPR screen in the human beta cell line EndoC-βH1 identified more than half of all protein networks that were also identified in a previous CRISPR screen assessing insulin content in a mouse insulinoma cell line, highlighting conserved interspecies regulatory networks [1]. While shared protein networks were identified in both CRISPR screens, only a small proportion of screening hits (7% or 43 genes) were overlapping, highlighting the importance of performing the screen in a human cellular model system but also potentially diverging experimental strategies. Of the 20 genes from our CRISPR screen that were overlapping predicted effector transcripts, only one gene and the primary phenotypic readout in both screens, *INS*, was also identified by the CRISPR screen in the mouse insulinoma cell line [1].

The reason many investigators select to work in rodent beta cells lines is one largely of experimental ease. Although the human beta cell line EndoC-βH1 demonstrates functional characteristics closely resembling human primary cell it is also associated with difficult growth and culture characteristics [2–4]. To obtain perturbation evidence in this physiologically relevant cellular model, we overcame the associated obstacles and variations across passages through highly consistent culturing conditions, integration of two independent replicates, stringent analysis parameters focusing on high reproducibility and rigorous proof of concept studies.

While many screening hits have not been studied in the context of human beta cell function so far, some genes have already been the focus of functional investigations albeit the involved mechanisms in T2D pathogenesis remain to be fully discovered. Whilst the effects of *TBC1D4* on T2D pathogenesis have been considered to be primarily associated with insulin resistance, the effect on insulin content observed in this screen is consistent with previous studies indicating a crucial role within beta cells[5–10]. The same protein GTPase network also contained *ARAP1*, a gene previously linked to T2D susceptibility adding another dimension to the interesting story at this locus where deletion or overexpression in mice has failed to detect an impact on beta cell function, pointing towards the nearby genes *STARD10* or *FCHSD2* as modulators of insulin secretion and mediators of disease risk at this GWAS locus[11–13].

In addition to its association with T2D risk based on GWAS and its intracellular role, CALCOCO2 has also been implicated in studies of the plasma proteome, demonstrating that reduced circulating levels of CALCOCO2 are associated with increased T2D risk [14,15]. Plasma proteins are key druggable targets and disease biomarkers, positioning CALCOCO2 at the center of investigations focusing not only on understanding the underlying functional mechanism of T2D but also potential therapeutic or diagnostic strategies [16].

1. Fang, Z. *et al.* Single-Cell Heterogeneity Analysis and CRISPR Screen Identify Key β-Cell-Specific Disease Genes. *Cell Rep.* **26**, 3132-3144.e7 (2019).

2. Grotz, A. K. *et al.* A CRISPR/Cas9 genome editing pipeline in the EndoC-βH1 cell line to study genes implicated in beta cell function. *Wellcome Open Res.* **4**, 150 (2019).

3. Hastoy, B. *et al.* Electrophysiological properties of human beta-cell lines EndoC-βH1 and -βH2 conform with human beta-cells. *Sci. Rep.* **8**, 1–16 (2018).

4. Lawlor, N. *et al.* Multiomic Profiling Identifies cis-Regulatory Networks Underlying Human Pancreatic β Cell Identity and Function. *Cell Rep.* **26**, 788-801.e6 (2019).

5. Bouzakri, K. *et al.* Rab GTPase-Activating Protein AS160 Is a Major Downstream Effector of Protein Kinase B/Akt Signaling in Pancreatic β-Cells. *Diabetes* **57**, 1195 LP – 1204 (2008).

6. Sakamoto, K. & Holman, G. D. Emerging role for AS160/TBC1D4 and TBC1D1 in the regulation of GLUT4 traffic. *Am. J. Physiol. Endocrinol. Metab.* **295**, E29-37 (2008).

7. Dash, S. *et al.* A truncation mutation in TBC1D4 in a family with acanthosis nigricans and postprandial hyperinsulinemia. *Proc. Natl. Acad. Sci. U. S. A.* **106**, 9350–9355 (2009).

8. Dash, S. *et al.* Analysis of TBC1D4 in patients with severe insulin resistance. *Diabetologia* **53**, 1239–1242 (2010).

9. Moltke, I. *et al.* A common Greenlandic TBC1D4 variant confers muscle insulin resistance

and type 2 diabetes. *Nature* **512**, 190–193 (2014).

10. Ndiaye, F. K. *et al.* Expression and functional assessment of candidate type 2 diabetes susceptibility genes identify four new genes contributing to human insulin secretion. *Mol. Metab.* **6**, 459–470 (2017).

11. Carrat, G. R. *et al.* Decreased STARD10 Expression Is Associated with Defective Insulin Secretion in Humans and Mice. *Am J Hum Genet* **100**, 238–256 (2017).

12. Hu, M. *et al.* Chromatin 3D interaction analysis of the STARD10 locus unveils FCHSD2 as a new regulator of insulin secretion. *bioRxiv* 2020.03.31.017707 (2020). doi:10.1101/2020.03.31.017707

13. Kulzer, J. R. *et al.* A Common Functional Regulatory Variant at a Type 2 Diabetes Locus Upregulates ARAP1 Expression in the Pancreatic Beta Cell. *Am J Hum Genet* **94**, 186–197 (2014).

14. Emilsson, V. *et al.* Co-regulatory networks of human serum proteins link genetics to disease. *Science (80-.).* **361**, 1–61 (2018).

15. Zheng, J. *et al.* Phenome-wide Mendelian randomization mapping the influence of the plasma proteome on complex diseases. *Nat. Genet.* **52**, 1122–1131 (2020).

16. Santos, R. *et al.* A comprehensive map of molecular drug targets. *Nat. Rev. Drug Discov.* **16**, 19–34 (2017).
