# Supplementary material for: A genome-wide CRISPR screen identifies CALCOCO2 as a regulator of beta cell function influencing type 2 diabetes risk
Source: Nat Genet. 2022 Dec 21;55(1):54–65. doi: 10.1038/s41588-022-01261-2 (PMC9839450; doi:10.1038/s41588-022-01261-2)
Supplement: Supplementary file 2 — Reporting Summary [file 41588_2022_1261_MOESM2_ESM.pdf]

Corresponding author(s): Anna Gloyn

Last updated by author(s): 17/10/22

Reporting Summary

Nature Research wishes to improve the reproducibility of the work that we publish. This form provides structure for consistency and transparency in reporting. For further information on Nature Research policies, see our Editorial Policies and the Editorial Policy Checklist.

Statistics

For all statistical analyses, confirm that the following items are present in the figure legend, table legend, main text, or Methods section.

n/a	Confirmed	
☐	☒	The exact sample size (*n*) for each experimental group/condition, given as a discrete number and unit of measurement
☐	☒	A statement on whether measurements were taken from distinct samples or whether the same sample was measured repeatedly
☐	☒	The statistical test(s) used AND whether they are one- or two-sided<br>*Only common tests should be described solely by name; describe more complex techniques in the Methods section.*
☐	☒	A description of all covariates tested
☐	☒	A description of any assumptions or corrections, such as tests of normality and adjustment for multiple comparisons
☐	☒	A full description of the statistical parameters including central tendency (e.g. means) or other basic estimates (e.g. regression coefficient) AND variation (e.g. standard deviation) or associated estimates of uncertainty (e.g. confidence intervals)
☐	☒	For null hypothesis testing, the test statistic (e.g. *F*, *t*, *r*) with confidence intervals, effect sizes, degrees of freedom and *P* value noted<br>*Give P values as exact values whenever suitable.*
☒	☐	For Bayesian analysis, information on the choice of priors and Markov chain Monte Carlo settings
☒	☐	For hierarchical and complex designs, identification of the appropriate level for tests and full reporting of outcomes
☐	☒	Estimates of effect sizes (e.g. Cohen's *d*, Pearson's *r*), indicating how they were calculated

Our web collection on statistics for biologists contains articles on many of the points above.

Software and code

Policy information about availability of computer code

Data collection	Commercial software associated with the following instruments was used to collect data:<br>- 7900HT Fast Real-Time PCR System (Applied Biosystems)<br>- EnSpire Alpha Plate Reader (Perkin Elmer)<br>- ChemiDoc MP Imaging System (Bio-Rad)<br>- Zeiss AxioM1 (Zeiss)<br>- FACSAria III (BD Biosciences)<br>- EM-1400 120kV (JEOL)<br>- STELLARIS 8 Confocal Microscope (Leica Microsystems).<br>- NEXTSeq500 (Illumina)<br>- JEM-1400 120kV (JEOL)
Data analysis	The following software/code was used to analyse the data:<br>- Flowjo 10.6 (BD Biosciences)<br>- MAGeCK (v0.5.9.2) algorithm (Li et al. 2014)<br>- Python 3.8<br>- R 3.5<br>- STRING v11 (Szklarczyk et al. 2019)<br>- STAR v2.7.9a (Liao et al. 2014)<br>- featureCounts (v2.0.1)<br>- DESeq2 (v1.26.0) (Love et al. 2014)<br>- Database for Annotation, Visualization and Integrated Discovery 6.8 (DAVID)<br>- Image Lab 6.0 software (Bio-Rad)<br>- ImageJ 1.52b<br>- Prism 8.1 (GraphPad Software)

Respective code is available at https://doi.org/10.5281/zenodo.7226348.

For manuscripts utilizing custom algorithms or software that are central to the research but not yet described in published literature, software must be made available to editors and reviewers. We strongly encourage code deposition in a community repository (e.g. GitHub). See the Nature Research guidelines for submitting code & software for further information.

Data

Policy information about availability of data

All manuscripts must include a data availability statement. This statement should provide the following information, where applicable:

- Accession codes, unique identifiers, or web links for publicly available datasets
- A list of figures that have associated raw data
- A description of any restrictions on data availability

Fastq sequencing files from the CRISPR screen have been deposited in the European Nucleotide Archive (ENA) at EMBL-EBI under accession number PRJEB44712. Fastq sequencing files from RNA-Sequencing experiments for siCALCOCO2 samples have been deposited in the European Genome-phenome Archive (EGA) under study number EGAS00001006127 and EndoC-βH1 expression data from a previously published study can be accessed under PRJEB15283 (ENA)78. The data is freely available to download while the processed counts can be found in the Supplementary Dataset 1. RNA-Seq data were aligned to the human genome reference GRCh38 (ftp://ftp.ensembl.org/pub/release-101/fasta/homo_sapiens/dna/Homo_sapiens.GRCh38.dna.primary_assembly.fa.gz) and counted with the gene annotation (ftp://ftp.ensembl.org/pub/release-101/gtf/homo_sapiens/Homo_sapiens.GRCh38.101.gtf.gz) downloaded from Ensembl database. Source data of unprocessed blots and Extended Data can be accessed online.

Field-specific reporting

Please select the one below that is the best fit for your research. If you are not sure, read the appropriate sections before making your selection.

☒ Life sciences ☐ Behavioural & social sciences ☐ Ecological, evolutionary & environmental sciences

For a reference copy of the document with all sections, see nature.com/documents/nr-reporting-summary-flat.pdf

Life sciences study design

All studies must disclose on these points even when the disclosure is negative.

Sample size	shRNA knockdown experiments in primary islets and functional assessment in T2D risk allele carriers were carried out in the maximum number of primary human islets preparations made available through the Integrated Islet Distribution Network (IIDP) and the Alberta Diabetes Institute Islet Core at the time.<br>Two CRISPR screening replicates were performed based on previous assessment of the screening performance with varying replicate numbers which determined two replicates as ideal replicate to return ratio (Hart et al. 2017).<br>Effect sizes could not be estimated prospectively (precluding power analysis) for cellular experiments in EndoC-βH1, hence standard scientific convention of performing three independent experiments was followed (three independent passages of cells with three technical replicates each). Based on previous experience with the technical variation that can be assumed in those methods, these sample sizes were deemed sufficient to detect real biological effects. For some experiments that allowed for simultaneous sample collection across multiple independently set up experiments such as insulin content, multiples of three were performed with the analysis only being performed after all data had been collected.
Data exclusions	Significant outlier in primary islet assessments from T2D risk carriers were excluded based on the pre-established ROUT outlier test (Q=1%). sgRNA in the CRISPR screen with low read counts of less than 10 and sgRNAs mapping to genes that were not expressed in EndoC-βH1 were excluded from the analysis. Data was also not included if experimental mistakes occurred and were noted while performing the experiment.
Replication	All functional cellular experiments were reliably reproduced in at least three independent passages. Of those experiments, effects in insulin content were reproduced in two different laboratories and by several independent researchers. For the human islet data, replication on the same samples could not be attempted due to the difficulties in obtaining primary islets from cadaveric donors.
Randomization	The islets from human donor were grouped by genotype, so randomization was not required. For cellular experiments, samples were grouped by treatment such as control and siCALCOCO2 and randomly positioned on microplates to minimize any possibly systematic bias from technical artefacts. Other randomization was not required in this observational study.
Blinding	Assessors of subjective EM quantification experiments were blinded to treatments (siNT or siCALCOCO2) in data analysis. Data collection was performed with a blinded investigator present to ensure unbiased collection of images. Blinding was not required in other experiments as the outcomes were not based on subjective measurement.

Reporting for specific materials, systems and methods

We require information from authors about some types of materials, experimental systems and methods used in many studies. Here, indicate whether each material, system or method listed is relevant to your study. If you are not sure if a list item applies to your research, read the appropriate section before selecting a response.

April 2020

Materials & experimental systems

n/a	Involved in the study	
☐	☒	Antibodies
☐	☒	Eukaryotic cell lines
☒	☐	Palaeontology and archaeology
☒	☐	Animals and other organisms
☐	☒	Human research participants
☒	☐	Clinical data
☒	☐	Dual use research of concern

Methods

n/a	Involved in the study	
☒	☐	ChIP-seq
☐	☒	Flow cytometry
☒	☐	MRI-based neuroimaging

Antibodies

Antibodies used

Western Blot Antibodies
β-Tubulin 1 in 2000 Mouse monoclonal Santa Cruz, sc-365791 (E10)
GAPDH 1 in 10000 Rabbit polyclonal Abcam, ab37168
INS 1 in 1000 Mouse monoclonal Santa Cruz, sc-393887 (C-12)
C-peptide 1 in 1000 Mouse monoclonal ExBio, 11-247-C100 (C-PEP-01)
CALCOCO2 1 in 1000 Mouse monoclonal Santa Cruz, sc-376540 (F6)
PCSK1 1 in 1000 Rabbit polyclonal Proteintech, 28219-1-AP
PCSK2 1 in 1000 Rabbit polyclonal Proteintech, 10553-1-AP
Anti-mouse IgG HRP 1 in 2500 Rabbit polyclonal Thermo Fisher, 31460
Anti-rabbit IgG HRP 1 in 2500 Goat polyclonal Thermo Fisher, 31450

Immunofluorescence Antibodies (Pancreas sections)
CALCOCO2 1 in 100 Mouse monoclonal Santa Cruz, sc-376540 (F6)
INS 1 in 300 Guinea-pig polyclonal Dako, A0564
Anti-mouse IgG AF555 1 in 300 Donkey polyclonal Invitrogen, A-31570
Anti-guinea-pig IgG AF647 1 in 300 Goat polyclonal Abcam, ab150187

Immunofluorescence Antibodies (EndoC-βH1)
CALCOCO2 1 in 100 Rabbit polyclonal Abcam, ab68588
LC3 1 in 500 Rabbit polyclonal Novus Biologicals, NB100-2220
INS 1 in 400 Guinea-pig polyclonal Progen, 16049
Anti-mouse IgG AF555 1 in 300 Donkey polyclonal Invitrogen, A-31570
Anti-rabbit IgG AF488 1 in 300 Donkey polyclonal Invitrogen, A-21206
Anti-guinea-pig IgG AF647 1 in 300 Donkey polyclonal JacksonImmuno, 2340476

FACS antibodies
INS 1 in 10 Rabbit monoclonal Cell Signaling, #3014 (C27C9)
INS 1 in 10 Rat monoclonal DSHB, GN-ID4
INS 1 in 10 Rat monoclonal R&D, MAB1417 (# 182410)
IgG 1 in 10 Rabbit monoclonal Cell Signaling, #3900 (DA1E)
IgG 1 in 10 Rat Invitrogen, 02-9602
Anti-rat IgG-AF488 1 in 500 Chicken polyclonal Invitrogen, A-21470
Anti-rabbit IgG-AF488 1 in 200 Goat polyclonal Invitrogen, A-11034

Validation

All antibodies are commercially available and were validated by the manufacturer. Specifically, the antibodies used for functional experiments targeting INS and CALCOCO2 were validated using siRNA knockdown, CRISPR KO, INS-negative cell lines and siRNA knockdown, respectively.

β-Tubulin Santa Cruz, sc-365791 (E10): Validated by manufacturer (expected size in WB).
GAPDH Abcam, ab37168: Validated by manufacturer (expected size and localization in WB, IHC-P, ICC/IF)
INS Santa Cruz, sc-393887 (C-12): Validated by manufacturer (expected size and localization in WB, ICC/IF)
C-peptide ExBio, 11-247-C100 (C-PEP-01): Validated by manufacturer (expected size and localization in ELISA, RIA, IHC(P), ICC)
CALCOCO2 Santa Cruz, sc-376540 (F6): Validated through loss of detected protein in WB at expected size upon silencing of CALCOCO2 (Fig 4d)
PCSK1 Proteintech, 28219-1-AP: Validated by manufacturer (expected size and localization in WB, IHC,ELISA)
PCSK2 Proteintech, 10553-1-AP: Validated by manufacturer (expected size in WB)
INS Dako, A0564: Validated through loss of detected protein in IF in tissue not expressing INS (exocrine) (ED Fig 7b)
CALCOCO2 Abcam, ab68588: Validated through loss of detected protein in IF upon silencing of CALCOCO2 (ED Fig 7a)
LC3 Novus Biologicals, NB100-2220: Validated by manufacturer (KO validated in WB, ICC/IF)
INS Progen, 16049: Validated by manufacturer (expected localization in ICC/IF and IHC)
INS Cell Signaling, #3014 (C27C9): Validated through loss of signal for protein in FACS upon silencing of INS and no signal in cell line not expressing INS (Fig 1b-f,ED Fig 1)
INS DSHB, GN-ID4: Validated through loss of signal for protein in FACS upon silencing of INS (ED Fig S1)
INS R&D, MAB1417 (# 182410): Validated through loss of signal for protein in FACS upon silencing of INS (ED Fig S1)
IgG Cell Signaling, #3900 (DA1E): Validated by manufacturer, Isotype control antibodies (expected size in IP)

IgG Invitrogen, 02-9602: Validated by manufacturer, Isotype control antibodies

Eukaryotic cell lines

Policy information about cell lines

Cell line source(s)	- The EndoC-βH1 cell line was acquired from Raphael Scharfmann (EndoCells, Paris) under an MTA.<br>- HEK293T has been purchased from sigma, ref: 12022001.
Authentication	The EndoC-βH1 has not been authenticated by formal short tandem repeat (STR) profiling, but the cell line is routinely subjected to rigorous testing in a number of different ways, all of which have confirmed the identity as a bona fide human beta cell line. Relevant tests include microarray-based genotyping, RNA-seq, ATAC-seq, and extensive cellular phenotyping, including insulin secretion and content (see Grotz et al. 10.12688/wellcomeopenres.15447.2.) and electrophysiology (see Hastoy et al, 10.1038/s41598-018-34743-7).<br>HEk293T has been authenticated by the manufacturer through STR profiling.
Mycoplasma contamination	Cells were tested for mycoplasma contamination on a quarterly basis, and tested negative on all occasions.
Commonly misidentified lines (See ICLAC register)	No commonly misidentified cell lines were used in this study.

Human research participants

Policy information about studies involving human research participants

Population characteristics	Characteristics of human islet donors have been provided in Supplementary Table S6.
Recruitment	All donors' families gave written, informed consent for the use of pancreatic tissue in research and were not financially compensated. Islets were distributed through the Integrated Islet Distribution Network (IIDP) and the Alberta Diabetes Institute Islet Core.
Ethics oversight	Human pancreatic islets were isolated from deceased donors under ethical approval obtained from the Human Research Ethics Board of the University of Alberta (Pro00013094, Pro00001754).

Note that full information on the approval of the study protocol must also be provided in the manuscript.

Flow Cytometry

Plots

Confirm that:

☒ The axis labels state the marker and fluorochrome used (e.g. CD4-FITC).

☒ The axis scales are clearly visible. Include numbers along axes only for bottom left plot of group (a 'group' is an analysis of identical markers).

☒ All plots are contour plots with outliers or pseudocolor plots.

☒ A numerical value for number of cells or percentage (with statistics) is provided.

Methodology

Sample preparation	EndoC-βH1 cells were harvested and incubated with LIVE/DEAD Fixable Far Red Dead Cell Stain (Thermo Fisher) for 30 min at room temperature to distinguish live from dead cells and washed in 1% BSA in PBS. The cells were fixed and permeabilized using the BD Cytofix/Cytoperm kit (BD Biosciences) for 20 min at 4°C and washed using Perm/Wash Buffer (BD Biosciences). Staining with primary antibodies was performed overnight at 4°C followed by incubation with suitable secondary antibodies diluted in Perm/Wash Buffer. The samples were filtered through a 70 µm cell strainer and sorted on a FACSAria III (BD Biosciences) using a 100 µm nozzle. Isotype and transduction controls stained with each antibody alone were analyzed alongside the samples.
Instrument	Data was collected on a FACSAria III (BD Biosciences).
Software	Flow cytometry data was collected on commercial software associated with the FACSAria III (BD Biosciences) and analyzed using Flowjo 10.6 (BD Biosciences).
Cell population abundance	Values are described for a representatice replicate. FSC-A and SSC-A gating for cells indicated 92.8% cells, FSC-A and FSC-W gating indicated 88.4% singlets. Live/dead staining further gated for 74.9% cells. Sorted INS samples were reanalyzed on the FACSAria III with consistent parameters and demonstrated a purity of 99.1%.
Gating strategy	FSC-A/SSC-A gating was performed for an initial broad gating on cells, only debris with low values were excluded from the gate. FSC-A/FSC-W was applied to gate for single cells with exclusion of cells beyond the linear range. SSC-A/655nm gating was performed based on the live/dead staining and the distinct population of live cells with low 655nm values were sorted.

Insulin staining was assessed based on SSC-A/488nm and gates were determined based on the isotype control and control cells. The gating strategy is shown in ED Fig 2.

☒ Tick this box to confirm that a figure exemplifying the gating strategy is provided in the Supplementary Information.
