# Supplementary material for: A genome-wide CRISPR screen identifies CALCOCO2 as a regulator of beta cell function influencing type 2 diabetes risk
Source: Nat Genet. 2022 Dec 21;55(1):54–65. doi: 10.1038/s41588-022-01261-2 (PMC9839450; doi:10.1038/s41588-022-01261-2)
Supplement: Source Data Fig. 4 — Unprocessed western blots. [file 41588_2022_1261_MOESM5_ESM.pdf]

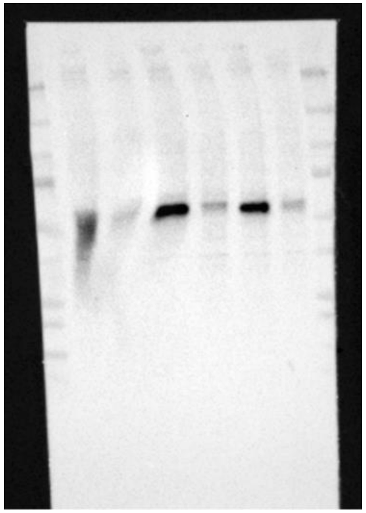

CALCOCO2

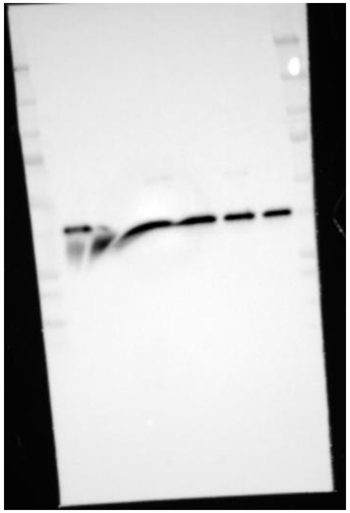

GAPDH

Figure 4d
Membrane probed with anti-CALCOCO2, washed and reprobed with anti-GAPDH.
Lane 5 and 6 are siNT and siCALCOCO2 samples as depicted in the manuscript.

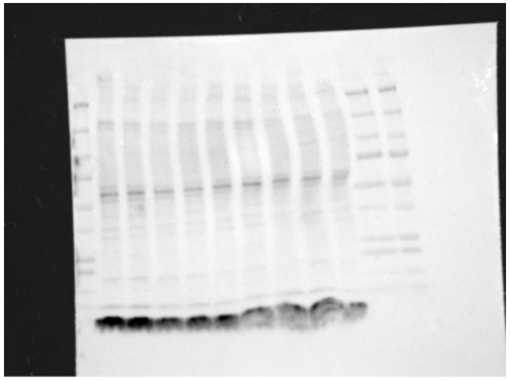

INS

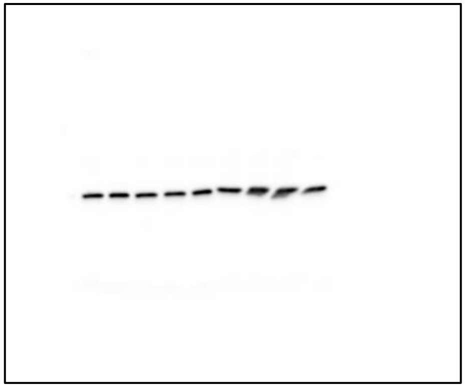

GAPDH

Figure 4h
Membrane probed with anti-INS, washed and reprobed with anti-GAPDH.
Lane 1 and 3 are siNT and siCALCOCO2 samples as depicted in the manuscript.
