# Supplementary material for: A genome-wide CRISPR screen identifies CALCOCO2 as a regulator of beta cell function influencing type 2 diabetes risk
Source: Nat Genet. 2022 Dec 21;55(1):54–65. doi: 10.1038/s41588-022-01261-2 (PMC9839450; doi:10.1038/s41588-022-01261-2)
Supplement: Source Data Extended Data Fig. 8 — Unprocessed western blots. [file 41588_2022_1261_MOESM6_ESM.pdf]

Figure 8n

Membrane probed with anti-PCSK1, washed, cut and reprobed with anti-GAPDH.
Lane 3 and 4 are siNT and siCALCOCO2 samples as depicted in the manuscript.

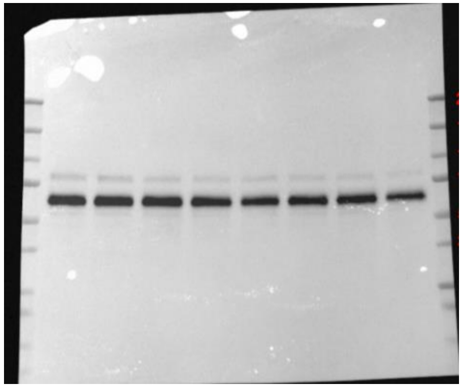

PCSK1

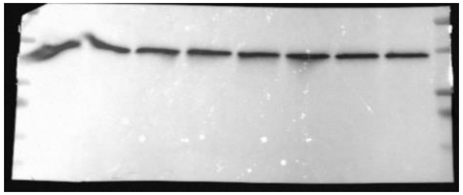

GAPDH

Figure 8o

Membrane probed with anti-INS, washed, cut and reprobed with anti-GAPDH.
Lane 5 and 6 are siNT and siCALCOCO2 samples as depicted in the manuscript.

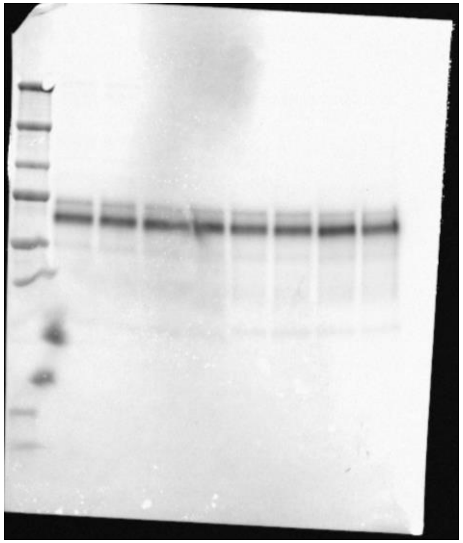

PCSK2

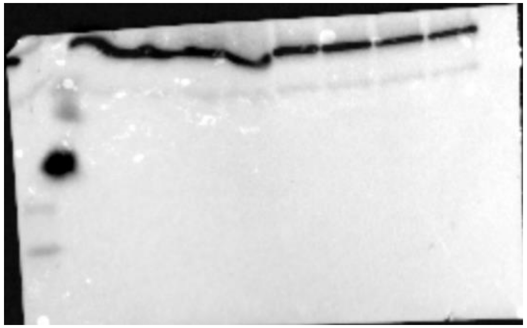

GAPDH
